# TAT-Mediated Transduction of MafA Protein In Utero Results in Enhanced Pancreatic Insulin Expression and Changes in Islet Morphology

**DOI:** 10.1371/journal.pone.0022364

**Published:** 2011-08-04

**Authors:** Nancy Vargas, Silvia Álvarez-Cubela, Jaime A. Giraldo, Margarita Nieto, Nicholas M. Fort, Sirlene Cechin, Enrique García, Pedro Espino-Grosso, Christopher A. Fraker, Camillo Ricordi, Luca Inverardi, Ricardo L. Pastori, Juan Domínguez-Bendala

**Affiliations:** 1 Diabetes Research Institute, University of Miami Leonard M. Miller School of Medicine, Miami, Florida, United States of America; 2 Department of Surgery, University of Miami Leonard M. Miller School of Medicine, Miami, Florida, United States of America; 3 Division of Endocrinology, Diabetes and Metabolism, Department of Medicine, University of Miami Leonard M. Miller School of Medicine, Miami, Florida, United States of America; 4 Department of Biomedical Engineering, University of Miami, Miami, Florida, United States of America; Ulm University, Germany

## Abstract

Alongside Pdx1 and Beta2/NeuroD, the transcription factor MafA has been shown to be instrumental in the maintenance of the beta cell phenotype. Indeed, a combination of MafA, Pdx1 and Ngn3 (an upstream regulator of Beta2/NeuroD) was recently reported to lead to the effective reprogramming of acinar cells into insulin-producing beta cells. These experiments set the stage for the development of new strategies to address the impairment of glycemic control in diabetic patients. However, the clinical applicability of reprogramming in this context is deemed to be poor due to the need to use viral vehicles for the delivery of the above factors. Here we describe a recombinant transducible version of the MafA protein (TAT-MafA) that penetrates across cell membranes with an efficiency of 100% and binds to the insulin promoter in vitro. When injected in utero into living mouse embryos, TAT-MafA significantly up-regulates target genes and induces enhanced insulin production as well as cytoarchitectural changes consistent with faster islet maturation. As the latest addition to our armamentarium of transducible proteins (which already includes Pdx1 and Ngn3), the purification and characterization of a functional TAT-MafA protein opens the door to prospective therapeutic uses that circumvent the use of viral delivery. To our knowledge, this is also the first report on the use of protein transduction in utero.

## Introduction

Maf proteins belong to a large class of transcription factors originally described as viral oncogenes [1]. They are characterized by the presence of a basic leucine zipper (b-Zip) domain and the ability to bind to DNA MARE (Maf Recognition Elements) either as homodimers or heterodimers with other b-Zip proteins. These transcription factors have been associated with the regulation of multiple differentiation processes, including hematopoiesis, skin and lens development and hind-brain segmentation [2], [3]. The best characterized Maf factors expressed in the pancreas are MafA and MafB [4]. Their role in pancreatic development has been difficult to ascertain, especially because their knockout has no overt effects in the specification of the major lineages of the organ [5], [6]. However, MafA −/− mice display glucose intolerance and develop age-dependent diabetes [5], and MafB knockouts exhibit some defects on endocrine cell maturation [6]. Since all Maf factors compete for the same MARE sites, their temporal and spatial pattern of expression is likely to affect developmental outcomes. Although MafB has also been recently shown to be essential for the appropriate regulation of Pdx1, Nkx6.1 and GLUT-2 in the final stages of islet β cell maturation [7], recent evidence suggests that a switch from MafB to MafA might be critical for the embryonic maturation and prolonged survival/function of β cells [4].

MafA has been found to selectively bind to the C1 (human)/RIPE3b (rat) element of the insulin gene promoter of β cells [8]. This sequence is of fundamental importance in the regulation of glucose-dependent insulin secretion [9]. While MafA is not a strong transactivator of the insulin promoter by itself, a synergistic action with Pdx1 and NeuroD/Beta2 has been demonstrated [10]. These two other factors are not exclusive of β cells, but this particular combination (Pdx1, NeuroD/Beta2 and MafA) is. Therefore, it has been hypothesized that the β cell-restricted expression of insulin is dictated by the concerted action of these three factors [11]. Perhaps not surprisingly, their ectopic expression in hepatocytes (which are ontogenically and physiologically related to β cells [12]) resulted in the activation of insulin expression [13]. More recently, a similar combination of genes (Pdx1, MafA and Ngn3, an upstream regulator of NeuroD/Beta2 [14]) also resulted in the *in vivo* reprogramming of pancreatic exocrine cells into β cells [15].

In addition to the regulation of insulin secretion, MafA may also be involved in other β cell processes by directly regulating genes such as prohormone convertase 1/3 (PC1/3), the glucagon-like peptide 1 receptor (GLP1-R), the glucose transporter GLUT-2, glucokinase, pyruvate carboxylase and the subunits Kir6.2 and SUR1 of potassium channels, as well as the transcription factors Nkx6.1, NeuroD/Beta2 and Pdx1 [16].

From a translational point of view, the emerging role of this gene as a potential therapeutic target for diabetes has not gone unnoticed. MafA is now acknowledged as a key element in most reprogramming strategies for islet β cell neogenesis [15], [17], [18]. However, as the use of viral vehicles to deliver reprogramming factors is considered unsafe in the context clinical therapies [19], [20], the quest for non-viral alternatives is a timely one. One such alternative is protein transduction, a technology by which membrane-permeable domains (protein transduction domains, or PTDs) enable recombinant proteins to efficiently penetrate into cells. One of the best studied PTDs is the 11-aa peptide derived from the basic domain of the TAT/HIV transactivator protein [21]. TAT is bound by charged heparan sulfate chains of cell membrane proteoglycans, taken up by macropinocytosis and then released to the cytoplasm [22], [23]. Due to its ease of engineering and effectiveness, TAT and other synthetic cationic relatives [24] have been used extensively to deliver full-length functional proteins both *in vitro* and *in vivo* [21]. Our team has been among the pioneers of protein transduction for pancreatic islet applications [25], [26], [27], [28], [29], [30], [31], [32], [33]. We have also previously described the generation of TAT-Ngn3, a transducible version of the pro-endocrine transcription factor that choreographs pancreatic endocrine cell specification. The use of this protein on pancreatic progenitor cells *in vitro* resulted in the targeted stimulation of α and β cell differentiation according to the developmental stage of the cells [34]. Here we report the purification and use of recombinant TAT-MafA in a variety of biological systems, including live developing mouse embryos onto which the protein was injected intracardially by ultrasound guidance techniques developed by our team (Nieto and Pastori, unpublished results). The functionality of the protein in the experimental group was evidenced by a significant increase of MafA target genes (chiefly insulin), as well as by changes in islet cytoarchitecture at birth. These findings are discussed in the context of the potential use of TAT-MafA not only as an important tool for developmental studies, but also as a valuable addition to our reprogramming armamentarium for therapeutic purposes.

## Results

### TAT-MafA effectively transduces living cells in vitro

**Figure 1:**
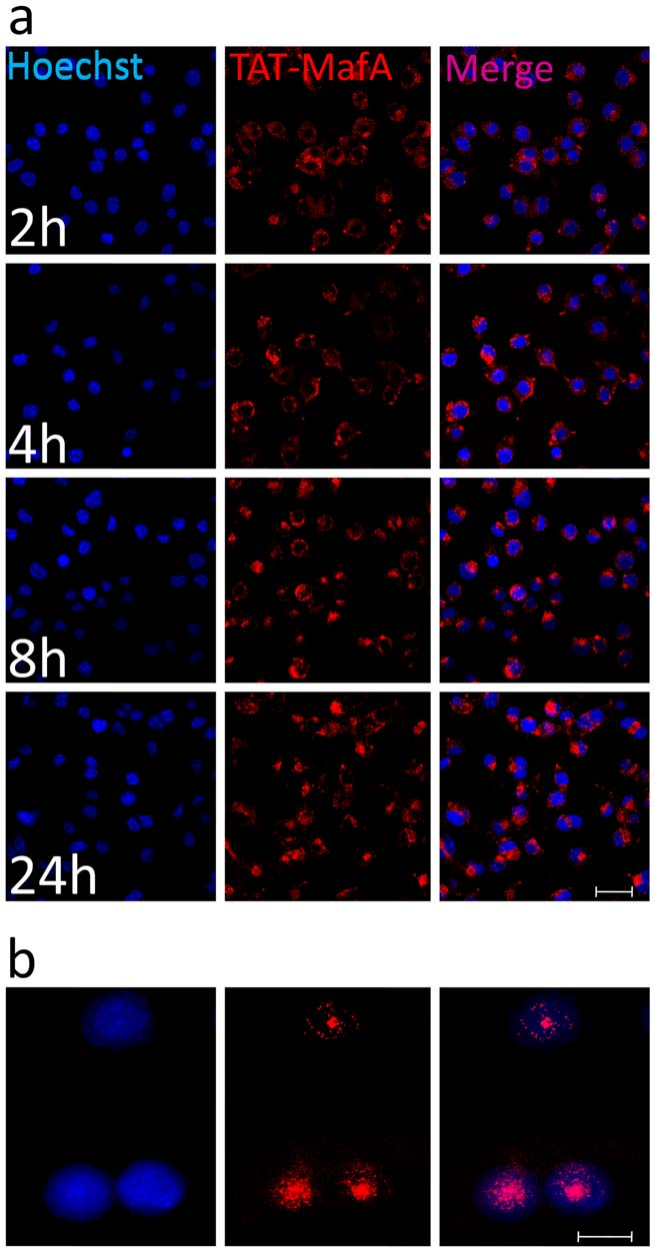
TAT-MafA uptake in β-TC3 cells. (A)TAT-MafA protein (1 µg/µl) was labeled with Alexa Fluor 568 and added to the culture medium of β-TC3 cells grown in glass bottom dishes (P35G-1.5-20-C 35 mm, MatTek Corporation, Ashland, MA) to ensure maximal optical clarity for live confocal microscopy. Hoescht (blue) was used as nuclear counter-staining (first column). An overlay of both channels is seen in the last column. The culture medium was removed and cells washed in PBS prior to each observation in order to eliminate free extracellular immunofluorescence background. Disturbance of the cultures prior to the time of observation was prevented by plating each individual group (time point) independently. Size bar: 20 µm. (B) Detail of nuclear localization of the protein after 8 h by live confocal microscopy (staining as above). Size bar: 5 µm.

In order to test whether TAT confers MafA the ability to penetrate across cellular membranes, mouse pancreatic insulinoma cells MIN-6 and β-TC3 were incubated with Alexa Fluor 568-labeled protein (final concentration in culture medium: 1 µM). Hoechst was used as a nuclear counter-staining in living cells. Figure 1a shows a time course experiment (2–24 h) using the latter cell line. In agreement with our previous experience with other TAT-fused proteins, virtually 100% of the cells were transduced as early as 2 h after addition of the labeled protein to the medium. Most of the TAT-MafA staining remains in cytoplasmic macropinocytotic vesicles at 24 h [23], but nuclear co-localization of released TAT-MafA was also demonstrated by confocal microscopy (figure 1b). This was particularly evident at later time points. MIN-6 cells displayed similar uptake kinetics, although in this cell line overt transduction could not be seen until 4 h of incubation (data not shown). The stability of the protein over longer incubation periods was not studied. The above observations indicate that TAT-MafA can be efficiently transduced *in vitro*.

### Recombinant TAT-MafA binds to the insulin promoter

**Figure 2:**
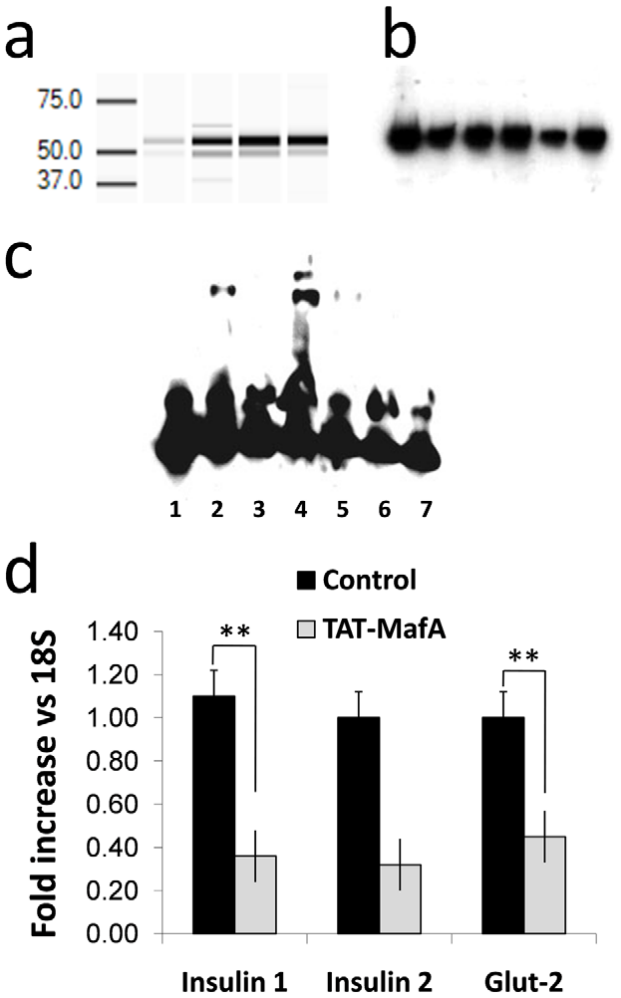
In vitro activity of TAT-MafA. (A) Experion™ virtual run of the last the purest eluate of TAT-MafA (far right), preceded by the three previous ones. (B) Western blot (MafA antibody) was used to confirm the identity of the purified protein. (C) Electrophoretic Mobility Shift Assay (EMSA). All samples include biotin-labeled DNA. 1. Labeled DNA alone (baseline control); 2. TAT-MafA (no competitor); 3. TAT-MafA+unlabeled DNA competitor (control); 4. Kinase treated TAT-MafA; 5. Kinase treated TAT-MafA+unlabeled DNA competitor (control); 6. Kinase alone (control); 7. Kinase+unlabeled DNA competitor (control) (D) qRT-PCR to measure relative expression of key pancreatic genes (insulin 1, insulin 2 and Glut-2) in e14.5 buds treated with TAT-MafA or vehicle. Black columns: control (vehicle); grey columns: TAT-MafA treatment. Bars: standard error.

TAT-MafA protein was purified as described in the Methods section. Optimization of the process results in high yield (>1 mg)-high purity (80–90%) protein. TAT-MafA can be detected as a ∼50 kD band by automated gel electrophoresis using a Experion bioanalyzer (Bio-Rad, Hercules, CA) (fig. 2a). The identity of the band was confirmed by Western blot using a rabbit polyclonal anti-MafA antibody (Abcam ab17976) (fig. 2b).

In order to determine whether TAT-MafA binds to its appropriate DNA target within the Insulin promoter, an electrophoretic mobility shift assay (EMSA) was done as indicated above. The sequence ATGGTCCGGAAATTGCAGCCTCAGCCCCCAGCCATC (−139 to −104 of the insulin promoter) was synthesized at Sigma-Aldrich (St. Louis, MO) for use in hybridization studies. This sequence encompasses the C1 box of the insulin promoter, a highly conserved region to which MafA is known to bind in a selective manner [35]. As mutational analysis has shown that the formation of MafA dimers capable of DNA binding is phosphorylation-dependent [36], [37], we used the ERK2 kinase (New England Biolabs, Ipswich, MA) to activate TAT-MafA before the reaction. As shown in fig. 2c, the use of phosphorylated TAT-MafA results in a significant mobility shift. With the exception of unphosphorylated TAT-MafA (which in our hands also exhibited basal DNA-binding activity), no other controls induced any band displacement.

### TAT-MafA has biological activity in vitro

A prediction of an effective binding to the insulin promoter *in vitro* would be an increase in insulin expression in relevant cell substrates. A set of experiments in which mouse insulinoma cells (β-TC3) were cultured for 24 h with the protein showed that purified TAT-MafA increased insulin expression by about 15-fold (data not shown). However, recent transgenic experiments indicate that ectopic expression of MafA too early during pancreatic development arrests the differentiation and proliferation of progenitor cells, possibly by inducing cyclin kinase inhibitors p27 and p57 [38]. A conclusion of these studies was that MafA may be used to enhance maturation, rather than specification, of β cells from their progenitors. We confirmed this observation by treating explanted e14.5 pancreatic buds with TAT-MafA for 48 h. Real-Time qRT-PCR analysis showed a significant down-regulation of the key β cell markers insulin 1, insulin 2 and GLUT-2 compared to vehicle-treated controls (fig. 2d). This down-regulation did not entail any loss in overall viability. In conclusion, purified TAT-MafA is able to recognize its biological DNA target and induce the expression of the insulin promoter, but has a detrimental effect on pancreatic progenitor cells at an early stage of development (e14.5). These results indicate that TAT-MafA is active and functions in a manner consistent with the predicted biological activity of the native protein.

### Targeting of the developing pancreas in utero by injection of transducible proteins

We decided to test the hypothesis that TAT-MafA has an effect on the maturation of late-stage pancreatic progenitors. However, unlike e14.5 pancreatic buds, late-stage fetal pancreata are large structures whose culture poses significant challenges in terms of appropriate oxygen and nutrient diffusion. Also, despite the known ability of TAT-fused proteins to go across relatively think structures (such as isolated islets), in our experience their penetration is rather limited beyond 300–400 microns *in vitro*. Because of the above considerations, the observation that we could not detect any significant difference at the gene expression level between TAT-MafA-supplemented and control e17.5 cultured pancreata was not surprising (Figure S1). However, the development of a novel intrauterine injection technique afforded us the possibility to study the role of TAT-MafA in a much more relevant *in vivo* model. In essence, using an ultrasound-guided approach, protein can be injected directly into the heart of embryos, from where it is distributed to all tissues, including the pancreas. This system offers a critical advantage over the *in vitro* setting, as the effects of the treatment can be observed without disturbing the native environment of the developing organ. Such advantage is especially significant for the study of late-stage pancreatic progenitors, because, as mentioned above, the large size of the pancreas at this time makes it difficult to establish viable organotypic cultures. This technology could also be potentially used in the context of human pre-natal therapy for a variety of conditions requiring *in utero* interventions (see Discussion).

**Figure 3:**
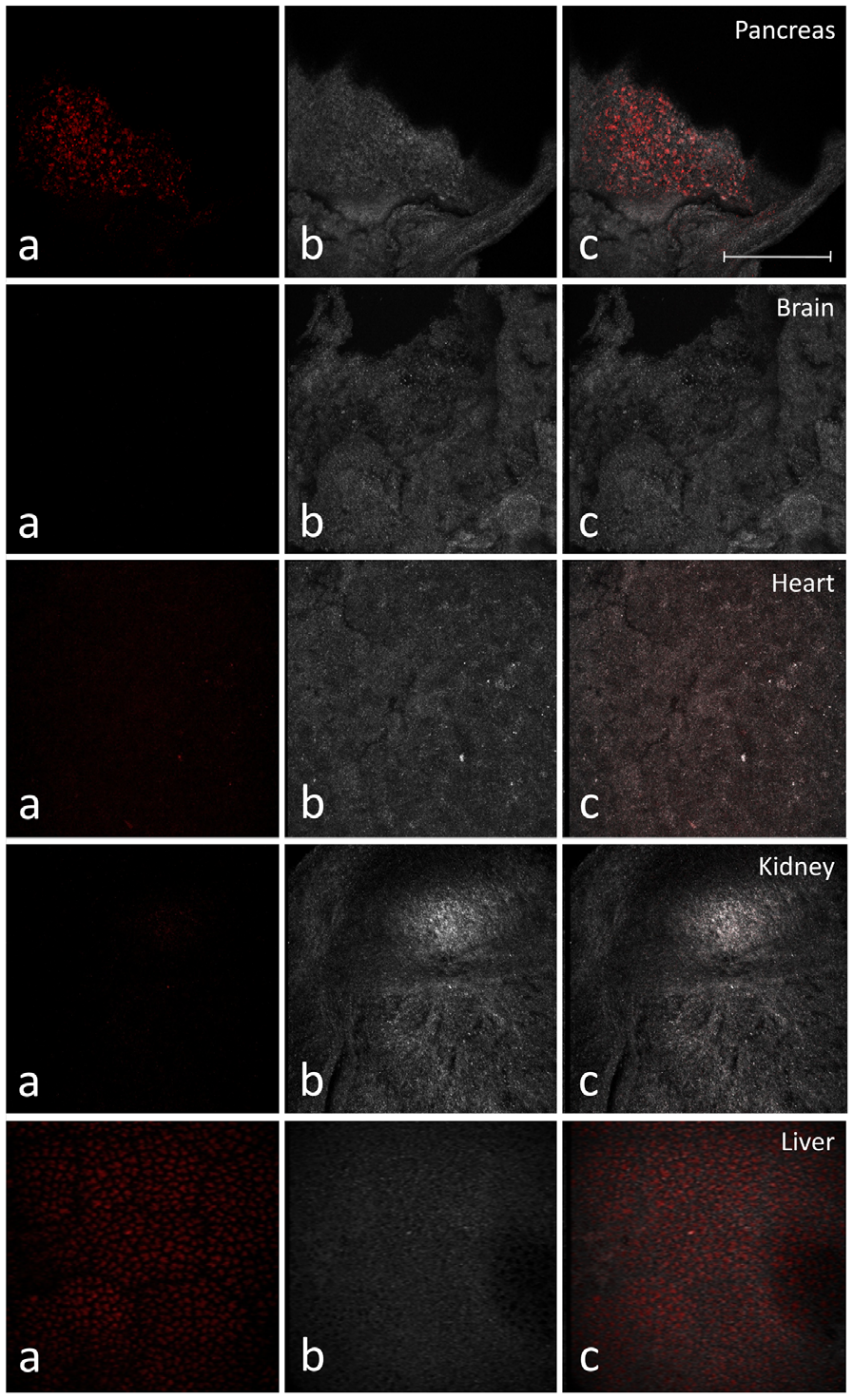
In vivo penetrability of TAT-fused proteins after in utero intra-cardiac injection. Examined organs are indicated. A, B and C represent Alexa Fluor 568-labeled TAT-βMafA (red), phase contrast of tissue, and composite image, respectively. Images were obtained 4 h after the injection by two-photon confocal microscopy. Size bar: 500 µm.

We used a labeled TAT-fused version of β-galactosidase (TAT-βgal) to establish the technique. Thus, Alexa Fluor 568-labeled TAT-βgal was injected directly into the heart of e17.5 embryos as described in Methods. Viability remains high and most embryos go to term normally (data not shown). In these preliminary experiments, the embryos were retrieved 4 h after the injection and their pancreata examined by multi-photon confocal microscopy. Figure S2 shows that the pervasion of TAT-βgal throughout the majority of the organ is quite evident. Next, we repeated these experiments with our protein of interest (TAT-MafA), also labeled in the same fashion. As shown in figure 3, TAT-MafA accumulates effectively both in the liver and the pancreas, but very little in other peripheral organs such as the brain, the kidney or the heart. We therefore demonstrate that intra-cardial injection allows for the relatively selective mobilization of the protein to the hepato-pancreatic region.

### Injection of TAT-MafA to developing embryos in vivo induces changes in pancreatic development

**Figure 4:**
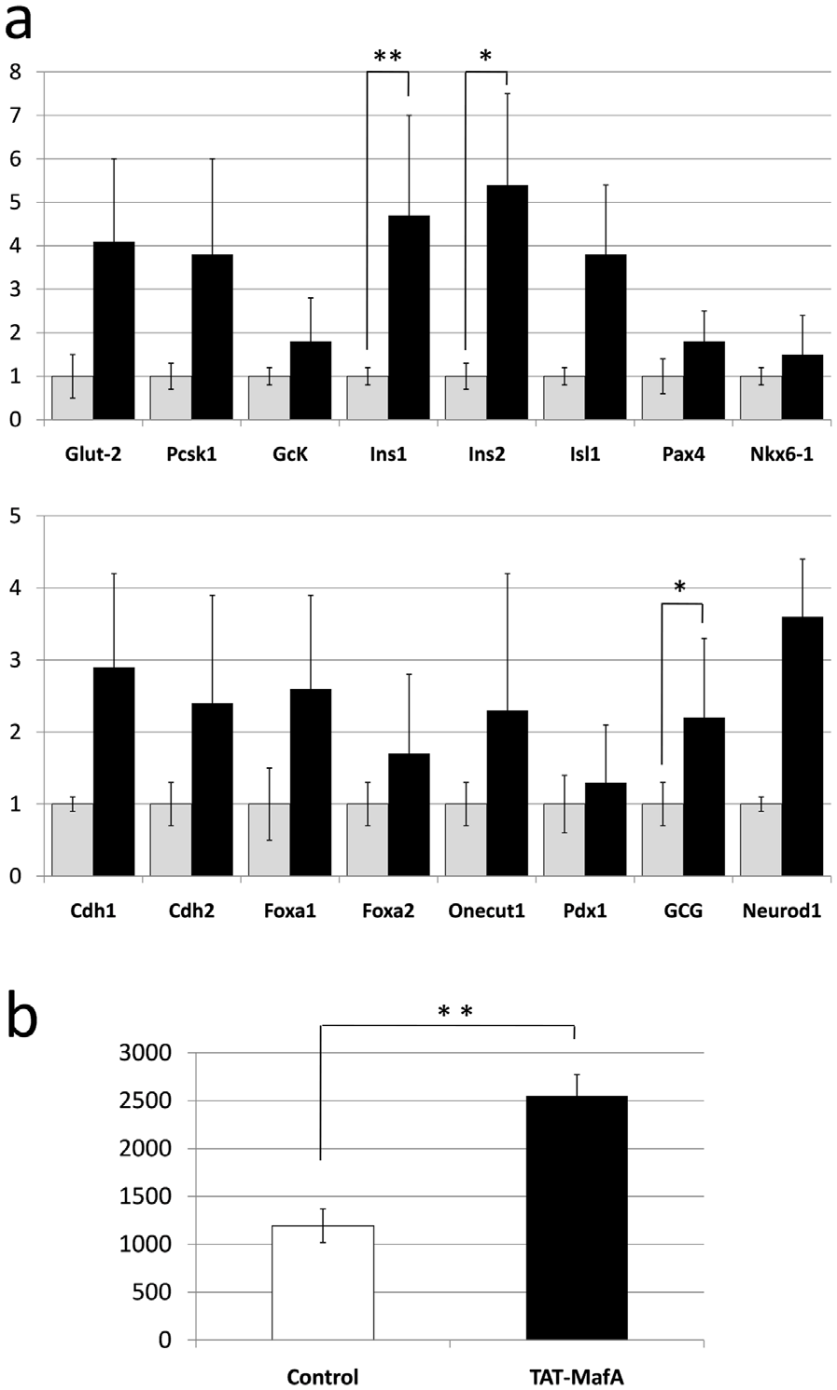
Gene and protein expression analysis of TAT-MafA-treated pancreata in neonate pups. (A) qRT-PCR of pancreatic markers in pancreata explanted from neonate pups. Grey columns: controls (embryos injected with vehicle). Black columns: TAT-MafA-treated embryos. Bars: standard error. Y axis: -fold increase over control ( = 1). (B) Total insulin extraction of pancreata explanted from neonate embryos injected *in utero* with vehicle (open column) or TAT-MafA (black column). Y axis: units of insulin (pM).

**Figure 5:**
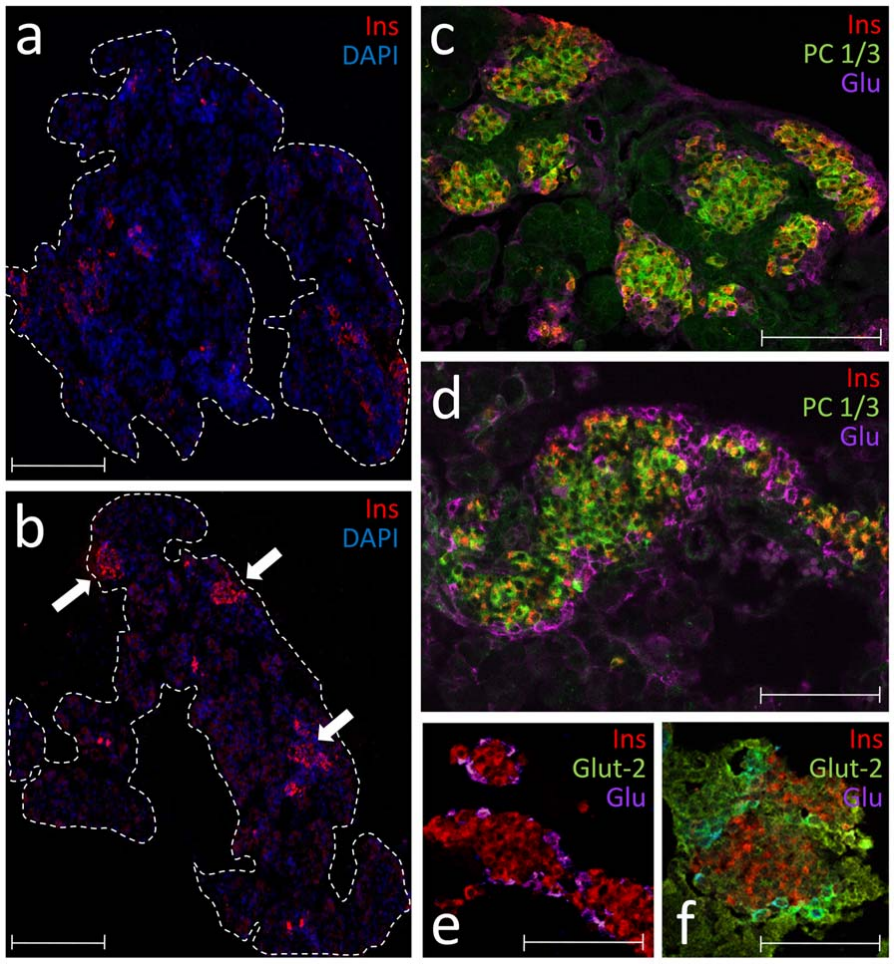
Histological analysis of TAT-MafA-treated pancreata in neonate pups. (A) Representative section of a control pancreas (*in utero* injection: vehicle), showing a disorganized pattern of β cell expression (red) in islets. (B) Representative section of a pancreas of a neonate pup treated *in utero* with TAT-MafA. Islets are larger, rounder and more organized than in controls. Size bars: 500 µm. (C–D) High magnification confocal microphotograph of control neonatal (C) and TAT-MafA-treated (D) pancreata. While the former exhibits small islets in the process of coalescence, islets from the latter are already larger in size. Prohormone convertase (PC) 1/3 staining (green) was similar in both samples despite a trend in favor of the experimental group by qRT-PCR (fig. 4). Glucagon staining (purple) was stronger in (D) at the same exposure. Size bars: 75 µm. (E) Glut-2 staining (green) is not evident in control samples, but can be observed in TAT-MafA samples (F). Size bars: 75 µm.

In order to test the above hypothesis, we injected e17.5 embryos with purified TAT-MafA and allowed them to go to term. Controls were injected with the same vehicle used for the protein. All the embryos retrieved from each mother (4 females/group, 7–8 embryos/female) were pooled and considered an individual *n* for statistical analyses. This sample size (*n* = 4) was determined to have adequate power to detect statistical differences with an alpha error level of 5% and beta error level of 50%. Upon birth, pups were sacrificed and their pancreata collected for further analysis. No significant differences could be observed either in the size or the gross anatomy of the embryos from each group. The pancreata were similar at the macroscopic level. Tissue samples were taken for qRT-PCR, insulin extraction and immunofluorescence (IF). As shown in figure 4a, the level of expression of all the pancreatic markers analyzed by quantitative real-time RT-PCR was elevated in the experimental group vs. the control. This increase was statistically significant for the endocrine cell markers glucagon (P = 0.0254), insulin 1 (P = 0.0043) and insulin 2 (P = 0.0363). The pancreatic content of insulin in the TAT-MafA-treated embryos doubled that found in controls, as measured by ELISA (*n* = 4; P = 0.0009) (figure 4b). IF analysis confirmed some of these findings. Islets from TAT-MafA-treated embryos are rounded and compact, with bright insulin and glucagon staining (figure 5b and d). Control pancreata, in contrast, exhibit smaller, more ragged and less organized islets, with weaker insulin and glucagon signal (figure 5a and c). No differences in proliferation were noted (data not shown). Immunostaining for prohormone convertase 1 (PC1/3, encoded by the Pcsk1 gene), an enzyme critically involved in the biosynthesis of insulin, appears to be similar in both groups despite a trend towards higher gene expression levels in the experimental group (figure 5c and d). However, staining for the glucose transporter 2 (Glut-2) could be observed in TAT-MafA-treated pancreata co-localizing with hormone-positive cells (figure 5f) but it was virtually undetectable in the control group (figure 5e). This is in agreement with the trends observed in the gene expression analyses.

## Discussion

We present here a novel combination of two techniques (protein transduction and *in utero* intracardial delivery) with a great potential both for the design of basic developmental research and human therapy. We have effectively purified and demonstrated the biological function of TAT-MafA, a transducible version of a protein that in recent years has garnered significant attention in the field of pancreatic development and reprogramming.

The flexibility afforded by TAT-MafA for the design of basic developmental studies is difficult to match by conventional transgenic strategies. Even with the caveat that there is a potential for off-target effects (although accumulation is largely hepato-pancreatic, the protein will be delivered to virtually every tissue), the route herein described opens new avenues of research that may complement, and even occasionally replace, experimental designs based on the generation of transgenic animals. Our findings on the effect of MafA on the e17.5 developing pancreas, for instance, would have otherwise required the cumbersome generation of transgenic mice with an inducible promoter. Our approach, in contrast, entailed only the ultrasound-guided intra-cardial injection of TAT-MafA at the desired developmental stage. Of note, as we have been able to inject embryos as small as e10.5 without significant loss of viability (Nieto *et al*, Nieto and Pastori, unpublished results), the applicability range of this technique spans organogenesis almost in its entirety.

Our observations clearly set the stage for more in-depth studies about the biological role of TAT-MafA in β cell specification. An obvious development of the present work, for instance, would be to examine functional parameters of islets from TAT-MafA-treated animals. However, since our primary objective was to demonstrate that TAT-MafA would work as predicted in the context of a novel delivery approach, such experiments were deemed to be beyond the scope of this study. Having said this, our findings are not only consistent with what is known about the function of the native gene, but also unveil some potential new functions that warrant additional study. Particularly intriguing is the observation that MafA may be associated with the process of islet coalescence and cytoarchitecture reshaping. It is known that neonatal islets typically exhibit a disorganized morphology, with less defined contours than those of adult origin [39]. While our controls adhere to that pattern, TAT-MafA injected embryos have larger, rounder and more adult-like islets. The molecular mechanism behind this phenomenon remains unclear, but it has been proposed that the acquisition of glucose-responsive insulin secretion requires an extensive remodeling of the islet cytoarchitecture affecting crucial regulatory events such as paracrine and cell-to-cell interactions [39]. At the molecular level, it has been shown that the maturation of β cells is accompanied by up-regulation of the expression of tight and adherens junction-associated proteins in islet cells [40], with quantifiable changes in the pattern of connexins, gap junction membrane density and coupling changes [41]. In this context, a simple explanation for our results would be that MafA would contribute to a faster islet remodeling just by accelerating β cell maturation, which is in fact its best-known function [4], [38]. Our own observation that functional β cell genes such as PC1/3, Glut-2 or GK are up-regulated upon TAT-MafA treatment would support this conclusion.

In addition to its well-documented role in the maturation of β cells, MafA has achieved –somewhat unexpectedly– notoriety as a key component of a triad of transcription factors that also include the better-known master regulators Pdx1 and Ngn3. The joint ectopic expression of these three factors in pancreatic acinar tissue results in their permanent reprogramming to insulin-producing β cells that could be used for the treatment of type 1 diabetes [15]. Although the clinical implications of such biotechnological feat were dampened by the need to use adenoviral vehicles, our findings timely complement the already reported generation of transducible Pdx1 [42] and Ngn3 [34] over the past few years. This opens the door to the controlled *in vitro* reprogramming of pancreatic exocrine tissue by means of protein transduction, a technique that completely circumvents the safety concerns posed by the use of viruses. The amount of tissue that is now routinely discarded after each clinical islet isolation (80–90% of which is arguably exocrine) would represent an invaluable potential source of insulin-producing cells. The sheer numbers of cells that could be cultured from such discards would make expansion almost unnecessary.

Our work also presents another significant innovation for the conduct of developmental biology studies, namely the *in vivo* intracardial injection of an agent suspected of having an effect on fetal ontogeny. Although there are previous reports describing *in utero* and fetal intracardial injection [43], [44], this is the first report on the use of bioactive transducible proteins. From a therapeutic perspective, the refinement of intra-cardial delivery techniques may also present distinct advantages over umbilical cord injection (typically hindered by poor placental transfer [45]) for a variety of potentially life-threatening prenatal conditions such as fetal cardiac arrhythmia [45], congenital adrenal hyperplasia [46] or fetal hemolytic disease [47], [48].

## Materials and Methods

### Protein subcloning and purification

The gene sequence of human MafA was optimized for expression in *E. coli* by Genscript (Piscataway, NJ) and then cloned into the multicloning site of pTAT-2.1 (pET28) expression vector (kindly given by S. Dowdy, UCSD) using *Sac*I and *Hind*III sites. BL21 *E. coli* bacteria were transformed with the plasmid, which also contains a 6xHis tag for affinity purification. Bacteria were cultured in LB medium with kanamycin (50 µg/ml) at 37°C to OD_600 nm_ = 0.8. Protein expression was induced with 1 mM IPTG (isopropyl β-D-thiogalactopyranoside) for 4 hours. After induction, the cells were harvested by centrifugation at 5,000 g for 15 min and washed with 1× PBS. The pellet was frozen at −80°C and subsequently resuspended in urea Buffer (6 M urea, 20 mM Hepes, 500 mM NaCL, 5 mM imidazole, pH 7.14) plus protease inhibitor (1 mM), lysozyme and benzonase. After incubation on ice (40 min), the pellet was sonicated (21 s pulse with 1 min between each pulse) and then centrifuged for 25 min at 12,000 g. Solubilized protein was purified by affinity chromatography on a 1 ml His-Trap column under denaturing conditions and elution in non-denaturing buffer. Chromatography was performed in three consecutive steps using a GE Healthcare Äkta purifier System (Waukeska, WI). First step: starting buffer (6 M urea, 20 mM Hepes, 500 mM NaCL, 5 mM imidazole, pH 7.14), end buffer (6 M urea, 20 mM Hepes, 500 mM NaCL, 100 mM imidazole, pH 7.14). Second step: starting buffer (6 M urea, 20 mM Hepes, 500 mM NaCL, 100 mM imidazole, pH 7.14), end buffer (20 mM Hepes, 500 mM NaCL, 100 mM imidazole, pH 7.14). Third step: starting buffer (20 mM Hepes, 500 mM NaCL, 100 mM imidazole, pH 7.14), elution buffer (20 mM Hepes, 250 mM NaCL, 750 mM imidazole, pH 7.14).

### Electrophoretic mobility shift assay (EMSA)

To study the DNA-binding specificity of TAT-MafA, we used the Pierce LightShift® Chemiluminescent EMSA Kit (Cat. 20148). DNA oligos were custom ordered from Sigma-Aldrich (St Louis, MO). Hybridization of complementary DNA chains was carried out according to the thermocycler method in Technical Tip # 45 from Thermo Scientific. To determine the proper concentration of labeled DNA, the 1 pM stock of biotin-labeled DNA was diluted to various concentrations, 20 ul of which were run in a Lonza PAGEr® Gold Precast Gel (Cat. 58525) at 100 V until the dye had migrated 2/3 of the way down the gel. The gel was then transferred to a nylon membrane and biotin-labeled DNA was detected by chemiluminiscence. A dilution of 200 fmol/µl was deemed optimal for imaging. Unlabeled DNA was used as a competitor at a concentration of 10 pmol/µl for the entire procedure. TAT-MafA phosphorylation was carried out by treatment with ERK2 kinase [49] (New England Biolabs, Ipswich, MA) according to the manufacturer's instructions. Protein was treated with kinase or PBS (control). In addition, kinase alone, where PBS was used instead of TAT-MafA protein, was also included as a control. These samples were subsequently used for preparation of the three main experimental EMSA binding reactions (PBS treated TAT-MafA; ERK2 kinase treated TAT-MafA; ERK2 kinase alone). 82.15 µg of TAT-MafA protein were used. Incubation with the kinase was done at 37°C for 1 h. During the incubation, the binding reactions were prepared from the Pierce EMSA Kit along with the three kit control reactions. Phosphorylation reaction products were then subjected to an additional 20 minute incubation for protein-DNA binding. 5 µl of loading buffer were added and samples run until the dye front was ¾ of the total length of the gel. Following transfer to a nylon membrane, the samples were cross-linked and analyzed by chemiluminiscence.

### In vitro culture of embryonic pancreatic buds and intrauterine embryo injection

All methods herein described involving the use of animals have been approved by the University of Miami IACUC. C57BL/6 mice (20–25 g body weight) were used for all animal experiments. Mating of the animals was set later in the day, and the following day the females were checked for vaginal plugs. Noon of that day was considered to be gestational time point e0.5. Pancreatic buds at e14.5 were explanted and cultured as described in [34]. For intrauterine embryo injection, pregnancy was confirmed by ultrasound imaging (Vevo 770, VisualSonics, Toronto, ON, Canada) at e17.5. At this time, pregnant females were anesthetized by inhalation of 2% isofluorane and placed on a preheated platform in the supine position with ECG electrodes taped to their legs to monitor the heart rate. Body temperature was maintained with the heating pad between 36°C and 38°C. Hair was removed from the abdomen using a chemical hair remover (Nair, Carter-Horner, Mississauga, ON, Canada). To provide a coupling medium for the transducer, a prewarmed ultrasound gel (Allegiance; Cardinal Health, McGaw Park, Illinois) was spread over the abdominal wall. For TAT-β-galactosidase injection experiments, protein (2 µg/ml) was labeled with Alexa Fluor 568 labeling kit (Invitrogen, Carlsbad, CA). Each visualized e17.5 embryo was injected intracardially with using a 500 µL syringe (Hamilton Company, catalogue No 81242, Reno, NV). The needle was guided using an ultrasound bio-microscope with B-mode image (Vevo 770, VisualSonics, Toronto, ON, Canada). Total volume of protein for each embryo was 10 µl. The mothers were sacrificed 4 h later and the pancreata of the embryos microdissected and examined using a multi-photon confocal microscope (Leica) to study the distribution of labeled TAT-β-galactosidase. For TAT-MafA experiments, seven to eight embryos per animal were injected with TAT-MafA (2.1 ug/µl) or protein vehicle (control). TAT alone was not used in controls because we and others have previously established that the TAT peptide is inert from a biological point of view [26], [50]. Pancreata were isolated for analysis immediately after birth (about e19.5–20.5).

### Quantitative Real-Time PCR

Total RNA was purified using Qiagen kits (QIAShredder, RNeasy and DNase-free). The First-Strand system (Roche, Basel, Switzerland) was used to generate cDNA (random oligomers). Relative gene expression was calculated using Taqman assays in either 7500 Fast, StepOne Plus or 7900 Real Time PCR cyclers (Applied Biosystems, Life Technologies, Carlsbad, CA). The latter was used to run custom-made Taqman® Low Density Microarray (TLDA) cards, which allow the simultaneous qRT-PCR analysis of up to 384 samples. The ΔCt method for relative quantification was deemed optimal for our application after discussion with Applied Biosystems researchers. All assays are designed to span exon-exon junctions, thus eliminating the possibility of genomic DNA contamination. qRT-PCR results are the average of several independent experiments. In addition, in each experiment each marker was analyzed in triplicates. Gene expression was normalized against 18S rRNA. This endogenous control has been validated in our system and proven extremely stable and more accurate than other standards.

### Total insulin extraction

Pancreata were lysed by ultrafreezing in the presence of 180 µl of T-PER (Thermo Scientific) and 20 µl of anti-protease (Roche), followed by physical maceration. Insulin was quantified by the LincoPlex endocrine panel kit (Linco-Invitrogen) using a Bioplex platform (Bio-Rad Laboratories, Inc).

### Western blots and immunofluorescence

Western blots were done according to standard methods [50]. The primary antibody for Maf-A was Abcam ab17976 (Cambridge, MA), used at a 1∶20,000 dilution. For immunofluorescence, embryonic pancreata were fixed in 4% paraformaldehyde overnight at 4°C and then embedded in OCT for 10 µm-thick sectioning. Staining was done with primary antibodies against anti-Prohormone Convertase PC1/PC3 (AB10553; Millipore, Billerica, MA) (1∶25 dilution), GLUT2 (ab85715; Abcam, 10 mg/mL), glucacon (A0565; Dako, Glostrup, Denmark) (1∶250 dilution) and insulin (A0564; Dako) (1∶250 dilution). Secondary antibodies: Donkey anti-rabbit 488, donkey anti-mouse 647 (both from Molecular Probes/Invitrogen, Carlsbad, CA) and DyLight 594 donkey anti-guinea pig (Jackson ImmunoResearch, West Grove, PA) (1∶400 dilution). Nucleus counter-staining was done with 4′,6-diamidino-2-phenylindole (DAPI) (St. Louis, MO). Images were captured using a Leica SP5 inverted confocal microscope with motorized stage (20× dry).

### Statistical analyses

Results are expressed as mean ± standard deviation (SD). The statistical significance of differences was assessed by the two-tailed Student's *t* test. In all comparisons, a value of *P*<0.05 was considered statistically significant. Sample size was determined by power analysis (5% alpha error level, 50% beta error level).

## Supporting Information

Figure S1 **Gene expression analysis of TAT-MafA-treated e17.5 pancreata.** (A) qRT-PCR of pancreatic markers in organs explanted from e17.5 embryos. Grey columns: controls (explants treated with vehicle). Black columns: TAT-MafA-treated explants. Bars: standard error. Y axis: -fold increase over control ( = 1). None of the differences is statistically significant.
(TIF)

Figure S2 **In vivo penetrability of TAT-fused proteins after in utero intra-cardiac injection.** (A–D) Alexa Fluor 568-labeled TAT-βgal is observed in the pancreas of embryos 4 h after the injection by two-photon confocal microscopy. (D) Control (pancreas of embryo injected with vehicle). Size bar: 75 µm.
(TIF)
