# Supplementary figures and images for: TAT-Mediated Transduction of MafA Protein In Utero Results in Enhanced Pancreatic Insulin Expression and Changes in Islet Morphology
Source: PLoS One. 2011 Aug 4;6(8):e22364. doi: 10.1371/journal.pone.0022364 (PMC3150355; doi:10.1371/journal.pone.0022364)

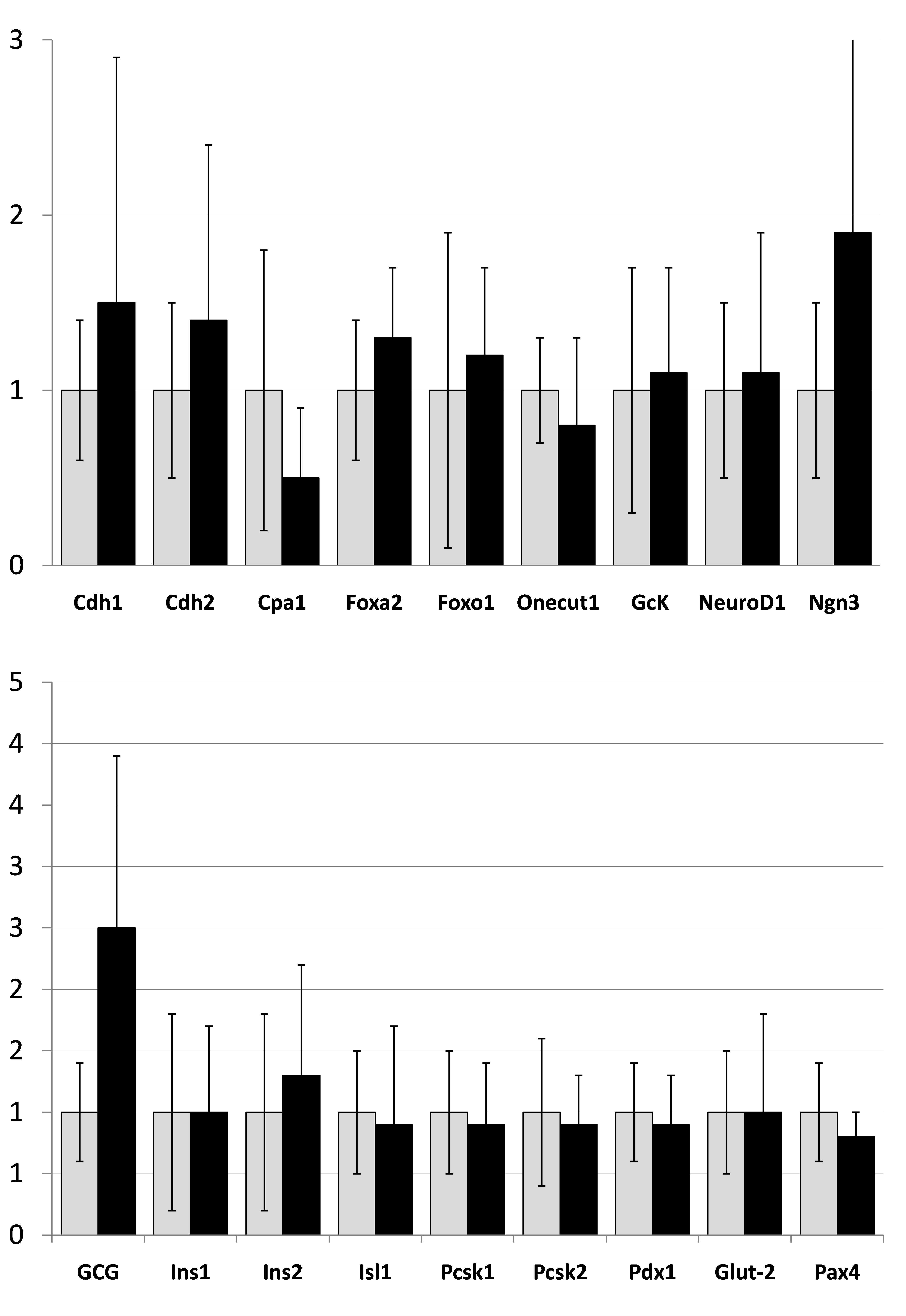

Supplement: Figure S1 — Gene expression analysis of TAT-MafA-treated e17.5 pancreata. (A) qRT-PCR of pancreatic markers in organs explanted from e17.5 embryos. Grey columns: controls (explants treated with vehicle). Black columns: TAT-MafA-treated explants. Bars: standard error. Y axis: -fold increase over control ( = 1). None of the differences is statistically significant. (TIF) [file pone.0022364.s001.tif]

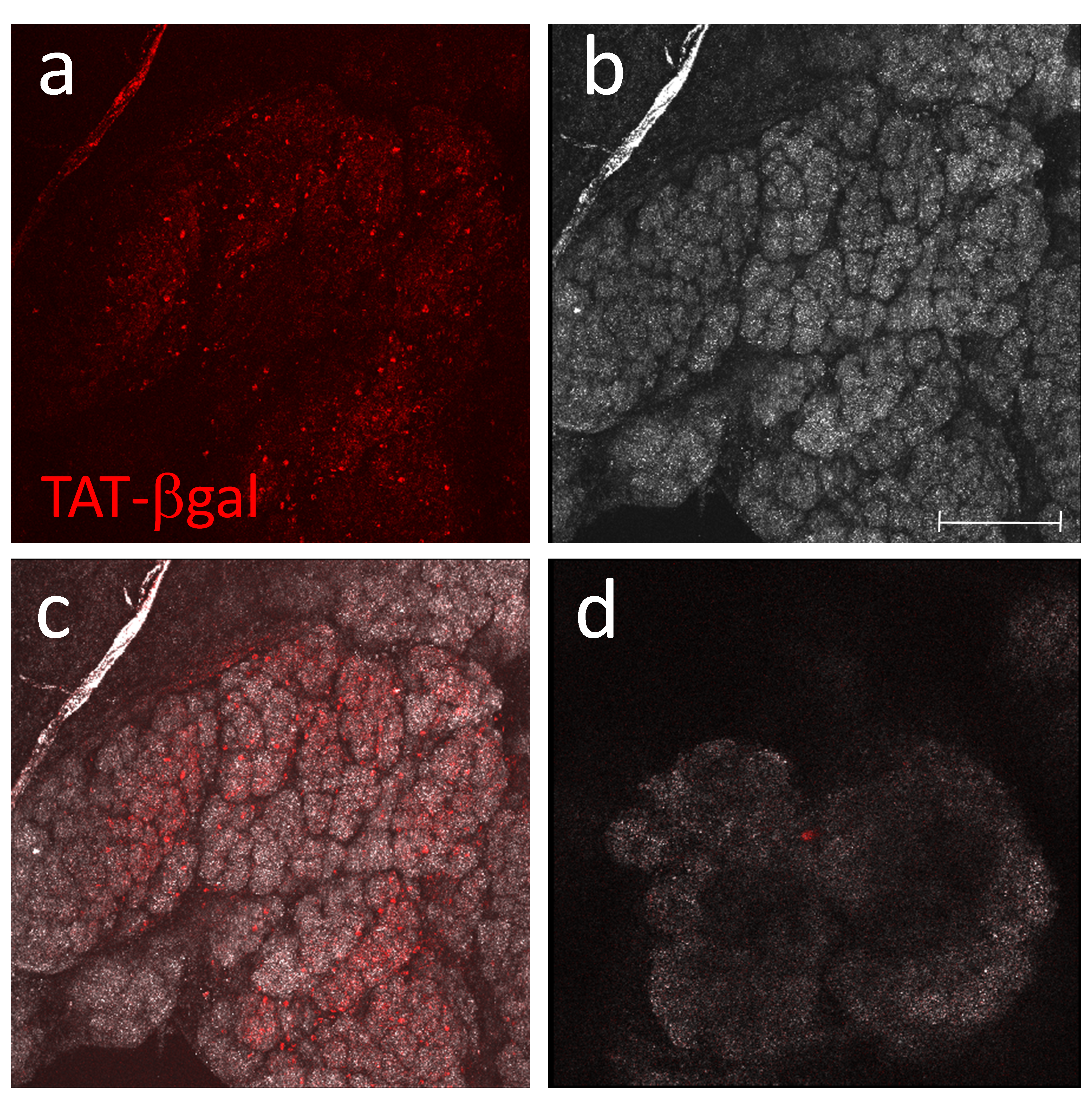

Supplement: Figure S2 — In vivo penetrability of TAT-fused proteins after in utero intra-cardiac injection. (A–D) Alexa Fluor 568-labeled TAT-βgal is observed in the pancreas of embryos 4 h after the injection by two-photon confocal microscopy. (D) Control (pancreas of embryo injected with vehicle). Size bar: 75 µm. (TIF) [file pone.0022364.s002.tif]
